# Optimal Algorithms for Improving Pressure-Sensitive Mat Centre of Pressure Measurements

**DOI:** 10.3390/s25051283

**Published:** 2025-02-20

**Authors:** Alexander Dawid Bincalar, Chris Freeman, m.c. schraefel

**Affiliations:** School of Electronics and Computer Science, University of Southampton, University Road, Southampton SO17 1BJ, UK; cf@ecs.soton.ac.uk (C.F.); mc+w@ecs.soton.ac.uk (m.c.s.)

**Keywords:** centre of pressure, optimisation, piezoresistive, pressure sensitive mat

## Abstract

The accurate measurement of human balance is required in numerous analysis and training applications. Force plates are frequently used but are too costly to be suitable for home-based systems such as balance training. A growing body of research and commercial products use Pressure-Sensitive Mats (PSMs) for balance measurement. Low-cost PSMs are constructed with a piezoresistive material and use copper tracks as conductors. However, these lack accuracy, as they often have a low resolution and suffer from noise, non-repeatable effects, and crosstalk. This paper proposes novel algorithms that enable the Centre of Pressure (CoP) to be computed using low-cost PSM designs with significantly higher accuracy than is currently achievable. A mathematical model of a general low-cost PSM was developed and used to select the design of the PSM (track width and placement) that maximises CoP accuracy. These yield new optimal PSM geometries that decrease the mean absolute CoP error from 17.37% to 5.47% for an 8 × 8 sensor layout. Then, knowledge of the footprint was used to further optimise accuracy, showing a decrease in absolute error from 17.37% to 3.93% for an 8 × 8 sensor layout. A third algorithm was derived using models of human movement to further reduce measurement error.

## 1. Introduction

Measuring balance and using it as real-time feedback in a training system has been shown to be an effective way to improve balance [1], as seen in the Biodex stability system [2] and Kinect and Wii balance board activities [3]. Such systems measure postural sway, either as a change in Centre of Pressure (CoP) or a change in the position of a physical or virtual marker on the body [4]. More generally, CoP is widely used in biomechanics as a quantitative measure of postural control and gait. It corresponds to the 2D projection of a person’s centre of mass on the floor, with changes over time used to assess stability. CoP is extensively used to, for example, predict the risk of falling in older adults [5], monitor trunk control in post-stroke hemiplegic patients [6], and monitor spontaneous movements in infants [7].

The gold standard for measuring balance through CoP is the force platform [8,9]. These are platforms held up by three or four load cells, which comprise strain gauges that accurately measure the forces applied to them. By comparing the difference in force readings from different load cells, the user’s CoP can be found, along with their mass [10]. Other ways to measure balance include camera methods, such as tracking the sway of points on the body through a depth camera like Microsoft Kinect [4,11]. Once calibrated, wearable inertial measurement units (IMUs) on multiple points of the body also assess balance by tracking accelerometry [12]. Pressure-Sensitive Mats (PSMs) also measure balance via CoP and have been used in balance studies [13,14,15], as well as many other applications covering vertical jump measurement, evaluating bodyweight exercise, sleep monitoring, and pressure sore prevention [16,17,18,19,20,21]. Instead of using load cells like a force plate, PSMs measure CoP by finding the average pressure location across a matrix of pressure sensors. These pressure sensors can be very thin (in the order of millimetres), allowing PSMs to have a narrow, lightweight, and flexible profile compared to force plates, which are rigid and several centimetres thick.

Low-cost balance measurement devices have many applications that would benefit people within society. Balance measurement devices are already used in balance rehabilitation or for general strength building, as balance training systems that feature CoP measurement have been found to improve balance in users [2]. In conjunction with Artificial Intelligence (AI), fall risk can be estimated [22], helping with the identification of those at risk. There is also the possibility to identify fatigue through balance measurement [23,24], which could be used in occupational health to prevent a worker from getting injured.

Current balance measurement devices like those described above are largely unwieldy in the aforementioned contexts: the bulk, weight, and cost of force plates, the special apparatus and space requirements for camera-based systems, the requirement to wear IMUs, and the high costs of commercial PSMs make accessing such potentially beneficial tools impractical for home use and even smaller clinical offices. In these contexts, a low-cost, low-profile, light, and flexible device that has a small footprint, requires little to no setup time, and sufficient accuracy would be ideal [25,26]. PSMs meet the low profile, flexibility, and accuracy requirements. However, current commercially available PSMs cost thousands to tens of thousands of pounds.

Since PSMs offer the most flexibility, have a small form factor, and are lightweight (a couple of hundred grams), researchers have investigated making lower-cost versions. Martinez and colleagues [27] presented a flexible PSM with a 16 × 16 resolution covering a 32 cm by 32 cm area using copper conductive tracks, a sheet of Velostat (a low-cost material that varies its resistance when force is applied), and a wireless MCU, costing less than GBP 100 in total. Saenz and colleagues [28] presented a PSM with a 32 × 32 resolution covering a 39 cm by 39 cm area. The downside to these low-cost PSMs are issues that decrease sensor accuracy and, hence, CoP accuracy, such as the sensing material making non-repeatable measurements and having low-accuracy, crosstalk noise that causes other sensors to read forces that are not there, as well as slow sensor settling times [29,30,31,32]. Numerous papers, however, have looked at algorithmic ways of solving these issues [27,31,32,33,34]. These existing approaches have focused on improving the accuracy of individual sensors, while our approach uniquely operates at a global level and optimises CoP measurement and overall PSM geometry using data from all sensors combined. By using this methodology, we show that such low-cost approaches to PSMs can be improved to give superior CoP accuracy.

Since CoP accuracy is lower in low-cost PSMs when compared to commercial high-resolution systems, we propose new techniques to improve CoP accuracy:

1. Using a non-uniform sensor layout: In the researched designs of [27,28], as well as all commercial systems, the sensor layouts are uniform; the sensors were the same size and had equal spacing between them. A uniform layout is not necessary, as most areas of the mat are unused during balance activities. Therefore, moving sensors from little-used areas to areas where they are highly used can increase CoP accuracy. This can be carried out in an optimal fashion using experimental data.
2. Fitting high-resolution profiles to low-resolution data: By using knowledge of footprint shape or footprint pressure profile (e.g., by previously measuring a high-resolution profile), the CoP accuracy obtained from a low-resolution PSM can be enhanced by fitting the higher quality data to the low-resolution data and then using the higher quality data to compute the CoP.
3. Smooth human movement: Because human motion is typically smooth and predictable (or can be predicted based on the task), models of human movement such as minimal jerk or minimal acceleration can also be embedded to remove the effects of noise and disturbance, further increasing CoP accuracy [35,36].

To implement our proposed optimisations for enhancing CoP accuracy, we developed what is, to the best of our knowledge, the first fully generalised mathematical model for simulating low-cost PSMs. Generated from our mathematical model, we present an ‘optimal’ sensor layout, which is a non-uniform layout where the spacing between sensors varies based on locations that experience more or less usage from the user. Three scenarios that reflect real balance tasks were optimised against, with the average CoP error from a standard uniform 8 × 8 mat over a 48 cm by 48 cm area being 17.37%. The CoP error when running the simulation scenarios on our optimal layout with the same resolution and dimensions became 5.47% in comparison. Through our mathematical model, we also test our “measured footprint” fitting optimisation, which also significantly reduced average CoP error down to 3.93% across the same simulations using the standard uniform layout. The largest advantage of the optimal geometry is that it does not add any additional cost or create any extra computational overhead when compared to implementing additional hardware or de-noising algorithms.

This paper provides several key contributions:

- The first mathematical model that fully describes the general form of a low-cost piezoresistive PSM.
- The development of three new optimisation algorithms to improve the design and accuracy of low-cost piezoresistive PSMs.
- When using our mathematical model and simulation scenarios, the average CoP error from an 8 × 8, 48 cm by 48 cm uniform mat layout is 17.37%.
- When using our optimal layout, the average CoP error became 5.47% for the same size and resolution mat.
- The measured footprint optimisation process has an average CoP error of 3.93% when performed with the simulation scenarios on the standard 8 × 8, 48 cm by 48 cm uniform layout.
- With our model and results, we now have new ways to produce better-performing, low-cost PSMs for applications ranging from rehabilitation assessments to in-home use.

The structure of the paper is as follows: Section 2 derives the low-cost PSM system model. Section 3 derives the approaches used to minimise CoP error through three proposed methods: optimising the layout of the PSM geometry, improving the CoP estimation through a known measured footprint, and using human movement models to further improve the previous optimisation. Section 4 introduces three balance scenarios that were used to test the optimisations. Section 5 links the previous three sections together and how they are used to generate our results. Section 6 contains the simulation results that use the PSM model in Section 2 and the optimisations in Section 3 to decrease the CoP error in the scenarios in Section 4. Section 7 discusses the practical implementation of the optimisations and the real-life considerations that need to be accounted for, as well as comparing the approaches with other techniques in the literature. Section 8 provides conclusions and avenues for future work.

## 2. Modelling a Pressure-Sensitive Mat

Our goal was to develop algorithms that produce a lower CoP error than a default uniform layout PSM with the same resolution and size. These were subsequently tested in common use-case scenarios: optimal geometry (Section 3.1), measured footprint (Section 3.2), and human smooth movement (Section 3.3).

To generate CoP error results from a uniform layout and our optimisation scenarios, it is first necessary to produce a generalised mathematical model that matches the behaviour of a piezoresistive PSM. To our knowledge, no such mathematical model currently exists, so it must first be derived. This model needs to compute the CoP from a matrix of pressure values, with the pressure values reflecting the results from a real piezoresistive sensor.

This mathematical model of the PSM can then predict the CoP values that would be produced by a real PSM of any geometry in response to a specified pressure profile. Therefore, this allows optimisations to be formulated that minimise the error between the ‘true CoP’ (e.g., measured from a high-resolution pressure profile) and the ‘estimated CoP’ from a low-resolution pressure profile. The optimal geometry finds the best set of pitch widths (the gap between sensors) that produces the lowest amount of CoP error. The measured footprint optimisation minimises the difference between the pressure of the previously measured high-resolution footprint and the low-resolution profile data by moving the high-resolution footprint around low-resolution data. The location of the high-resolution profile that produces the minimum difference in pressure is recorded, and then this location is used with the high-resolution data to compute the CoP.

When computing the CoP with the mathematical model, a pressure profile is required. The pressure profiles must represent how a user’s pressure changes when conducting real balance tasks, as this is the application of our PSM. We are minimising CoP error by assuming that the PSM is used for balance measurement; therefore, the scenarios used for the simulations have to be based on commonly used balance training tasks. To allow for multiple scenarios that vary with time, the average percentage CoP error across all time steps and all scenarios can be computed when calculating the optimal geometry.

The methods section continues below with a detailed mathematical derivation of the generalised PSM model. The derivation of the optimisations is covered in Section 3, and the application scenarios that vary the pressure profiles are given in Section 4. A list of the variables used in these derivations can be found in Appendix A.

### 2.1. Centre of Pressure

**Figure 1:**
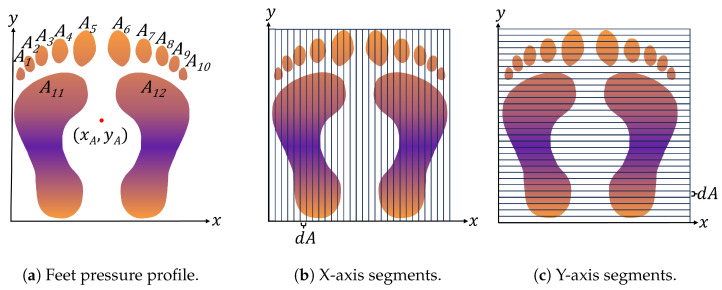
(**a**) A pressure profile of feet with Centre of Pressure $\left(x_{A},y_{A}\right)$. (**b**) The pressure profile, split into segments across the x-axis. (**c**) The pressure profile, split into segments across the y-axis.

Pressure is the force exerted per unit area due to contact with an object, and the Centre of Pressure (CoP) is the average location of the pressure within the overall contact area. For a given pressure profile, such as the pair of feet seen in Figure 1, with areas $A_{1},A_{2},\ldots ,A_{12}$ forming the overall area $A:=\cup_{i=1}^{12}A_{i}$ for a pressure profile P(x,y,t) at time *t*, the CoP for an axis can be found by segmenting the pressure profile into small strips of area dA (as shown in Figure 1).

The pressure in each segment is summed and multiplied by the segment’s location along the axis. The resulting values are then summed across all segments; dividing by the total pressure of the footprint, the CoP for that axis is given. The smaller the area segments, the more accurate the computation, with infinitesimally small segments giving the actual CoP. At time *t*, this has respective *x* and *y* components:

(1) $$x_{A}\left(t\right)=\frac{{\;\int \int \;}_{A}xP\left(x,y,t\right)dA}{{\;\int \int \;}_{A}P\left(x,y,t\right)dA},\;y_{A}\left(t\right)=\frac{{\;\int \int \;}_{A}yP\left(x,y,t\right)dA}{{\;\int \int \;}_{A}P\left(x,y,t\right)dA}$$

The discrete form of (1) appears in [37] and approximates the true value.

### 2.2. Pressure Measurement Using a PSM

A PSM approximates the pressure profile of an object or foot placed upon it, and it is constructed using conductive copper strips and a piezoresistive material such as Velostat, the resistance of which varies based on how much force is applied to the material. The Velostat is sandwiched between a criss-cross pattern of conductive strips, forming a matrix. Any pair of horizontal and vertical strips overlap to form a rectangular area termed a ‘sensor’. If a voltage is applied to the end of one strip and a pull-down resistor is applied to the other end, then the voltage at the latter end can be used to measure the Velostat resistance of the sensor. In practice, this is achieved using the analogue-digital converter (ADC) port of a microcontroller unit (MCU), and a general-purpose input/output (GPIO) is employed to select the strip using a multiplexor.

**Figure 2:**
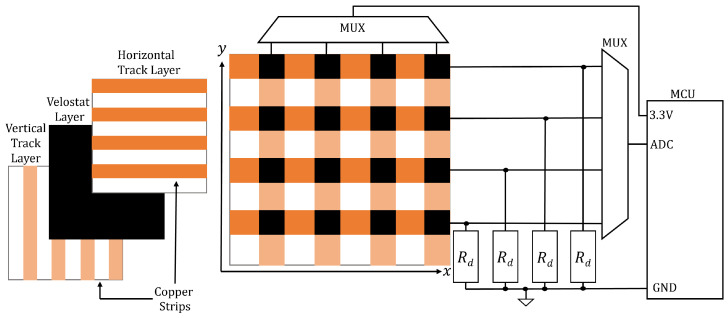
A diagram of a PSM and its associated hardware. Constitutive layers are shown on the left. The circuitry is shown on the right.

The MCU reads the ADC, computes the CoP, and/or sends the pressure data to another device for post-processing. Figure 2 shows a diagram of the typical hardware setup.

The next section defines the equations that govern the operation of a typical PSM, linking the applied pressure profile to the CoP measurement it produces.

### 2.3. PSM Model

**Figure 3:**
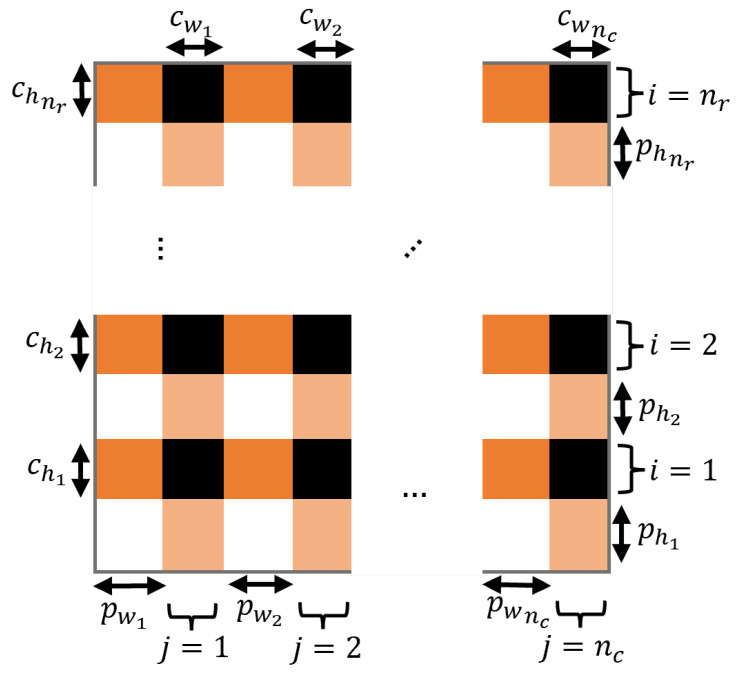
Pressure mat geometry with variable copper strip thickness and spacing in vertical and horizontal directions.

The most general pressure mat geometry is shown in Figure 3, in which the thickness of the conductive strip and the spacing between strips may change with position. Here, $n_{r}$ denotes the number of rows, $n_{c}$ denotes the number of columns, $c_{w_{i}}$ and $c_{h_{i}}$ denote the width and height of conductor *i*, and $p_{w_{i}}$ and $p_{h_{i}}$ denote the width and height between conductor *i* and i+1. Note that all existing PSM designs have uniform spacing, so these parameters have previously been fixed.

The sensing element i,j corresponds to the rectangular region where the horizontal strip *i* overlaps the vertical strip *j*. To compute the response of the PSM to an applied pressure profile, the midpoint position of the sensing region i,j is first defined as $\left(x_{j},y_{i}\right)$, with

(2) $$x_{j}=\frac{1}{2}c_{w_{j}}+\sum_{k=1}^{j-1}\left(c_{w_{k}}+p_{w_{k}}\right),\;y_{i}=\frac{1}{2}c_{h_{i}}+\sum_{k=1}^{i-1}\left(c_{h_{k}}+p_{h_{k}}\right)$$

**Figure 4:**
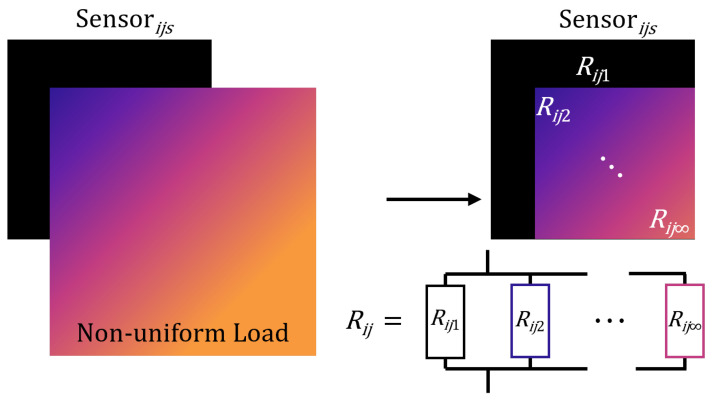
Geometry of an individual Velostat sensor, i,j, at position $\left(x_{j},y_{i}\right)$, experiencing a non-uniform pressure profile. The overall resistance is computed by dividing the area into regions and applying the parallel resistor relation.

The sensing region i,j is then divided into segments, as shown in Figure 4.

Denote a segment as ijs with area $A_{ijs}$ and observe that as the number of segments increases, the pressure applied to this segment tends to a constant value, denoted as $P_{ijs}$. The resistance across segment ijs is then also constant and given by the uniform pressure relation

(3) $$R_{ijs}=\frac{R_{0}}{R_{0}\kappa P_{ijs}+A_{ijs}}.$$

Here, κ and $R_{0}$ are constants, with specific values derived from [30].

The overall resistance of sensor i,j is then computed using the parallel resistor relation

(4) $$R_{ij}={\left(\frac{1}{R_{ij0}}+\frac{1}{R_{ij2}}+\cdots +\frac{1}{R_{ij\infty }}\right)}^{-1}={\left(\sum_{s=1}^{\infty }\frac{1}{R_{ijs}}\right)}^{-1}$$

where infinite segments are used to produce an exact value. Substituting (3) into (4) then yields (5), which expresses $R_{ij}$ in terms of $A_{ijs}$ and $P_{ijs}$ as

(5) $$R_{ij}=R_{0}{\left(\sum_{s=1}^{\infty }\left(R_{0}\kappa P_{ijs}+A_{ijs}\right)\right)}^{-1}=\frac{R_{0}}{R_{0}\kappa \sum_{s=1}^{\infty }\left(P_{ijs}\right)+\sum_{s=1}^{\infty }\left(A_{ijs}\right)}$$

This can then be simplified using the relation for total pressure over sensor i,j:

(6) $$\begin{array}{cc} P_{ij}:=\sum_{s=1}^{\infty }P_{ijs} & =\underset{A_{ij}}{\;\int \int \;}P\left(x,y\right)dA=P_{ij}\\ \; & =\int_{y_{i}-c_{h_{i}}/2}^{y_{i}+c_{h_{i}}/2}\int_{x_{j}-c_{w_{j}}/2}^{x_{j}+c_{w_{j}}/2}P\left(x,y\right)dxdy \end{array}$$

and its total area is the sum of all constituent segments so that

(7) $$A_{ij}:=\sum_{s=1}^{\infty }A_{ijs}=c_{h_{i}}c_{w_{j}}$$

Substituting (6) and (7) into (5) produces the overall resistance for sensor i,j:

(8) $$R_{ij}=\frac{R_{0}}{R_{0}k\int_{y_{i}-c_{h_{i}}/2}^{y_{i}+c_{h_{i}}/2}\int_{x_{j}-c_{w_{j}}/2}^{x_{j}+c_{w_{j}}/2}P\left(x,y\right)dxdy+c_{h_{i}}c_{w_{j}}}$$

Resistance, $R_{ij}$, is read by the MCU using a potential divider circuit, as shown in Figure 2. The raw value presented to the MCU is the sensor voltage:

(9) $$V_{ij}=V_{cc}\left(\frac{R_{d}}{R_{ij}+R_{d}}\right)$$

where $R_{d}$ is the divider resistance, and $V_{cc}$ is the common collector voltage. This is then quantised via a *b*-bit ADC that produces the final measured value:

(10) $${\bar{V}}_{ij}=\Delta \left\lfloor \frac{V_{ij}}{\Delta }\right\rfloor$$

using the quantisation step size, $\Delta =V_{cc}/\left(2^{b}-1\right)$, and floor operator, ⌊·⌋.

### 2.4. CoP Approximation Using a PSM

Having modelled the operation of existing PSM hardware, the computations used in PSM software were added to the model. First, each sampled voltage, ${\bar{V}}_{ij}$, value is converted back into a pressure reading by inverting (9) to obtain the quantised resistance:

(11) $${\bar{R}}_{ij}=R_{d}\left(\frac{V_{cc}}{{\bar{V}}_{ij}}-1\right)$$

which is substituted into (8) and rearranged to obtain the approximation of $P_{ij}$, given by

(12) $${\bar{P}}_{ij}=\frac{1}{R_{0}\kappa }\left(\frac{R_{0}}{R_{d}\left(\frac{V_{cc}}{{\bar{V}}_{ij}}-1\right)}-c_{h_{i}}c_{w_{j}}\right)$$

The set of measured pressure approximations, ${\left\{{\bar{P}}_{ij}\right\}}_{i=1,\cdots ,n_{r},\;j=1,\cdots ,n_{c}}$, are then used to approximate the true CoP values (1) by exchanging the area integral terms in (1) by their discrete approximations over the sensing elements, giving

(13) $$\begin{array}{cc} x_{E}\left(t\right) & =\frac{\sum_{i}^{n_{h}}\sum_{j}^{n_{w}}x_{j}{\bar{P}}_{ij}}{\sum_{i}^{n_{h}}\sum_{j}^{n_{w}}{\bar{P}}_{ij}}=\frac{\sum_{i}^{n_{h}}\sum_{j}^{n_{w}}\left(\frac{1}{2}c_{w_{j}}+\sum_{k=1}^{j-1}\left(c_{w_{k}}+p_{w_{k}}\right)\right){\bar{P}}_{ij}}{\sum_{i}^{n_{h}}\sum_{j}^{n_{w}}{\bar{P}}_{ij}},\\ \; & =\frac{\sum_{i}^{n_{h}}\sum_{j}^{n_{w}}\left(\frac{1}{2}c_{w_{j}}+\sum_{k=1}^{j-1}\left(c_{w_{k}}+p_{w_{k}}\right)\right)\left(\frac{R_{0}}{R_{d}\left(\frac{V_{cc}}{{\bar{V}}_{ij}}-1\right)}-c_{h_{i}}c_{w_{j}}\right)}{\sum_{i}^{n_{h}}\sum_{j}^{n_{w}}\left(\frac{R_{0}}{R_{d}\left(\frac{V_{cc}}{{\bar{V}}_{ij}}-1\right)}-c_{h_{i}}c_{w_{j}}\right)}, \end{array}$$

and

(14) $$\begin{array}{cc} y_{E}\left(t\right) & =\frac{\sum_{i}^{n_{h}}\sum_{j}^{n_{w}}y_{i}{\bar{P}}_{ij}}{\sum_{i}^{n_{h}}\sum_{j}^{n_{w}}{\bar{P}}_{ij}}=\frac{\sum_{i}^{n_{h}}\sum_{j}^{n_{w}}\left(\frac{1}{2}c_{h_{i}}+\sum_{k=1}^{i-1}\left(c_{h_{k}}+p_{h_{k}}\right)\right){\bar{P}}_{ij}}{\sum_{i}^{n_{h}}\sum_{j}^{n_{w}}\left(\frac{R_{0}}{R_{d}\left(\frac{V_{cc}}{{\bar{V}}_{ij}}-1\right)}-c_{h_{i}}c_{w_{j}}\right)},\\ \; & =\frac{\sum_{i}^{n_{h}}\sum_{j}^{n_{w}}\left(\frac{1}{2}c_{h_{i}}+\sum_{k=1}^{i-1}\left(c_{h_{k}}+p_{h_{k}}\right)\right)\left(\frac{R_{0}}{R_{d}\left(\frac{V_{cc}}{{\bar{V}}_{ij}}-1\right)}-c_{h_{i}}c_{w_{j}}\right)}{\sum_{i}^{n_{h}}\sum_{j}^{n_{w}}\left(\frac{R_{0}}{R_{d}\left(\frac{V_{cc}}{{\bar{V}}_{ij}}-1\right)}-c_{h_{i}}c_{w_{j}}\right)} \end{array}$$

Since all existing PSMs have uniform track spacing, these simplify to

(15) $$x_{E}\left(t\right)=\frac{\sum_{i}^{n_{h}}\sum_{j}^{n_{w}}\left(\frac{1}{2}c_{w}+\left(c_{w}+p_{w}\right)\left(j-1\right)\right)\left(\frac{R_{0}}{R_{d}\left(\frac{V_{cc}}{{\bar{V}}_{ij}}-1\right)}-c_{h}c_{w}\right)}{\sum_{i}^{n_{h}}\sum_{j}^{n_{w}}\left(\frac{R_{0}}{R_{d}\left(\frac{V_{cc}}{{\bar{V}}_{ij}}-1\right)}-c_{h}c_{w}\right)}$$

and

(16) $$y_{E}\left(t\right)=\frac{\sum_{i}^{n_{h}}\sum_{j}^{n_{w}}\left(\frac{1}{2}c_{h}+\left(c_{h}+p_{h}\right)\left(i-1\right)\right)\left(\frac{R_{0}}{R_{d}\left(\frac{V_{cc}}{{\bar{V}}_{ij}}-1\right)}-c_{h}c_{w}\right)}{\sum_{i}^{n_{h}}\sum_{j}^{n_{w}}\left(\frac{R_{0}}{R_{d}\left(\frac{V_{cc}}{{\bar{V}}_{ij}}-1\right)}-c_{h}c_{w}\right)}$$

This completes the model of a PSM: For a pressure profile P(x,y), the CoP approximations are given by (13), (14) based on the voltage measurements (8), (9) read by the PSM. Meanwhile, the true CoP co-ordinates are given by (1).

## 3. Optimisation of PSM Geometry and CoP Estimation

This section develops approaches to improve the CoP accuracy of existing PSMs. The first method, ‘Optimal PSM Geometry’ (Section 3.1), considers how the mat geometry (i.e., the parameters ${\left\{c_{w_{i}},c_{h_{j}},p_{w_{k}},p_{h_{l}}\right\}}_{i,j,k,l}$) should be selected to deliver greater accuracy. The second method, ‘CoP Estimation using Measured Footprint’ (Section 3.2), assumes the geometry has been selected and focuses on improving the accuracy of the CoP estimation algorithm. The third method, ‘CoP Estimation Using Human Movement Models’ (Section 3.3), can be used to enhance the previous optimisation method that uses the measured footprint by making the assumption that the acceleration or jerk is minimal during human movement.

To perform these optimisations, the general PSM model of Section 2 (below) was used alongside a set of pressure profiles that capture the intended movements. This set of pressure profiles may be experimentally collected or simulated using knowledge of the intended use case scenarios (see Section 4). Both approaches involve minimising the difference between the estimated CoP location of the PSM $\left(x_{E}\left(t\right),y_{E}\left(t\right)\right)$, given by (13), (14), and the real CoP location, given by (1).

This section will now go on to explain the mathematical derivations of the optimal geometry, measured footprint, and human movement model-optimisation methods.

### 3.1. Optimal PSM Geometry

To minimise CoP inaccuracy, the PSM geometry can be computed over a set of simulated or recorded pressure movements. For example, during balance exercises, the user places their feet in specific positions on the mat, meaning not all of the mat is used. As not all of the mat is utilised, sensors can be placed in higher densities in areas of the mat that will be used regularly and more sparsely in areas that are used less, boosting the resulting CoP accuracy.

To align with the practical construction of PSMs, we assume that a constant conductor width is employed for the sensors, and only the pitch widths and pitch heights between the sensors will be varied. Therefore, the optimisation problem involves finding the best set of pitch widths and pitch heights to give the lowest overall CoP error.

To define this optimisation problem, the unknown parameters of the PSM are first written as the vector

(17) $$\theta :=\left[c_{w_{1}},\cdots ,c_{w_{n_{c}}},c_{h_{1}},\cdots ,c_{h_{n_{r}}},p_{w_{1}},\cdots ,p_{w_{n_{c}}},p_{h_{1}},\cdots ,p_{h_{n_{r}}}\right].$$

Then, the optimisation problem is formulated as the minimisation of the error 2-norm, i.e.,

(18) $$\min_{\theta }J\left(\theta \right),\;J\left(\theta \right)=\int_{0}^{T}\left({\left(x_{E}\left(t\right)-x_{A}\left(t\right)\right)}^{2}+{\left(y_{E}\left(t\right)-y_{A}\left(t\right)\right)}^{2}\right)dt$$

where *T* is the duration of the pressure profile (or appended set of profiles). If it is desired that the overall PSM width and height be fixed at values *w* and *h*. respectively, the constraint

(19) $$\theta {\left[\begin{array}{cccccccccccc} 1 & \cdots & 1 & 0 & \cdots & 0 & 1 & \cdots & 1 & 0 & \cdots & 0\\ 0 & \cdots & 0 & 1 & \cdots & 1 & 0 & \cdots & 0 & 1 & \cdots & 1 \end{array}\right]}^{⊤}=\left[w,h\right]$$

is added to (18). Additional terms to, for example, penalise the relative cost of materials can also be added to the minimisation problem.

To solve minimisation (18), which minimises CoP error, it is necessary to substitute the PSM model Equations ((8)–(10)) and ((12)–(14)) into J(θ), yielding

(20) $$\begin{array}{cc} J(\theta )= & \int_{0}^{T}\left({\left(\frac{\sum_{i}^{n_{h}}\sum_{j}^{n_{w}}\left(\frac{1}{2}c_{w_{j}}+\sum_{k=1}^{j-1}\left(c_{w_{k}}+p_{w_{k}}\right)\right)\int_{y_{i}-c_{h_{i}}/2}^{y_{i}+c_{h_{i}}/2}\int_{x_{j}-c_{w_{j}}/2}^{x_{j}+c_{w_{j}}/2}\tilde{P}\left(x,y,t\right)dxdy}{\sum_{i}^{n_{h}}\sum_{j}^{n_{w}}\int_{y_{i}-c_{h_{i}}/2}^{y_{i}+c_{h_{i}}/2}\int_{x_{j}-c_{w_{j}}/2}^{x_{j}+c_{w_{j}}/2}\tilde{P}\left(x,y,t\right)dxdy}-x_{A}\left(t\right)\right)}^{2}\right.\\ \; & +\left.{\left(\frac{\sum_{i}^{n_{h}}\sum_{j}^{n_{w}}\left(\frac{1}{2}c_{h_{i}}+\sum_{k=1}^{i-1}\left(c_{h_{k}}+p_{h_{k}}\right)\right)\int_{y_{i}-c_{h_{i}}/2}^{y_{i}+c_{h_{i}}/2}\int_{x_{j}-c_{w_{j}}/2}^{x_{j}+c_{w_{j}}/2}\tilde{P}\left(x,y,t\right)dxdy}{\sum_{i}^{n_{h}}\sum_{j}^{n_{w}}\int_{y_{i}-c_{h_{i}}/2}^{y_{i}+c_{h_{i}}/2}\int_{x_{j}-c_{w_{j}}/2}^{x_{j}+c_{w_{j}}/2}\tilde{P}\left(x,y,t\right)dxdy}-y_{A}\left(t\right)\right)}^{2}\right)dt \end{array}$$

Solving the minimisation problem, therefore, only requires the simulated/measured pressure profile $\tilde{P}\left(x,y,t\right)$ defined over 0≤t≤T. The solution can be computed using one of many available constrained non-linear optimisation packages or via a brute force combinatorial search.

If the pressure profile is simulated, then $\tilde{P}\left(x,y,t\right)$ can be explicitly defined, allowing the integrals to be performed analytically. If $\tilde{P}\left(x,y,t\right)$ is measured, then an infinite resolution (i.e., continuous form) is not possible, and the term must be replaced by a discrete form that corresponds to the available measurement resolution. The case of using discrete-form data is addressed next.

#### Efficient Solution Form

To efficiently solve (20) using measured data (or to avoid analytic solutions in simulation), it is necessary to sample the pressure data $\tilde{P}\left(x,y,t\right)$ at the high-resolution positions X={0,Δx,2Δx,…,w}, Y={0,Δy,2Δy,…,h} and time instants T={0,Δt,2Δt,…,T}. This discrete form of $\tilde{P}\left(x,y,t\right)$ directly corresponds to a recorded pressure profile on a high-resolution PSM. Then, (1) is replaced by the high-resolution approximation

(21) $$x_{A}\left(t\right)=\frac{\sum_{y\in Y}\sum_{x\in X}x\tilde{P}\left(x,y,t\right)\Delta x\Delta y}{\sum_{y\in Y}\sum_{x\in X}\tilde{P}\left(x,y,t\right)\Delta x\Delta y},\;y_{A}\left(t\right)=\frac{\sum_{y\in Y}\sum_{x\in X}y\tilde{P}\left(x,y,t\right)\Delta x\Delta y}{\sum_{y\in Y}\sum_{x\in X}\tilde{P}\left(x,y,t\right)\Delta x\Delta y}$$

and (20) is similarly approximated by

(22) $$\begin{array}{cc} J(\theta )= & \sum_{t\in T}\left({\left(\frac{\sum_{i}^{n_{h}}\sum_{j}^{n_{w}}\left(\frac{1}{2}c_{w_{j}}+\sum_{k=1}^{j-1}\left(c_{w_{k}}+p_{w_{k}}\right)\right)\sum_{y\in Y\cap Y_{i}}\sum_{x\in X\cap X_{j}}\tilde{P}\left(x,y,t\right)\Delta x\Delta y}{\sum_{i}^{n_{h}}\sum_{j}^{n_{w}}\sum_{y\in Y\cap Y_{i}}\sum_{x\in X\cap X_{j}}\tilde{P}\left(x,y,t\right)\Delta x\Delta y}-x_{A}\left(t\right)\right)}^{2}\right.\\ \; & +\left.{\left(\frac{\sum_{i}^{n_{h}}\sum_{j}^{n_{w}}\left(\frac{1}{2}c_{h_{i}}+\sum_{k=1}^{i-1}\left(c_{h_{k}}+p_{h_{k}}\right)\right)\sum_{y\in Y\cap Y_{i}}\sum_{x\in X\cap X_{j}}\tilde{P}\left(x,y,t\right)\Delta x\Delta y}{\sum_{i}^{n_{h}}\sum_{j}^{n_{w}}\sum_{y\in Y\cap Y_{i}}\sum_{x\in X\cap X_{j}}\tilde{P}\left(x,y,t\right)\Delta x\Delta y}-y_{A}\left(t\right)\right)}^{2}\right)dt \end{array}$$

where $Y_{i}=\left[y_{i}-c_{h_{i}}/2,y_{i}+c_{h_{i}}/2\right]$, $X_{j}=\left[x_{j}-c_{w_{j}}/2,x_{j}+c_{w_{j}}/2\right]$. Clearly, as Δx,Δy,Δt→0, (22) converges to the true value (20). These terms are then substituted into the minimisation problem (18), replacing the previous terms (13), (14), and (1). Then, (22) is minimised to yield the optimal geometry of the low-cost PSM.

### 3.2. CoP Estimation Using Measured Footprint

In balance tasks where the base of support is fixed or where the base of support slides across the PSM, the pressure profiles are composed of components that maintain a fixed shape throughout the motion, with only their position or pressure changing over time. We can, therefore, use these fixed shape parameters to improve CoP computation accuracy. This technique requires having previously accurately measured these ‘footprint’ shapes and then solving an optimisation to find their most likely position given the measured data.

Let the set of known footprint areas be denoted ${\left\{A_{k}\left(x,y\right)\right\}}_{k=1,\ldots ,n_{k}}$, each defined at an arbitrary position in the PSM co-ordinate system. Then, any subsequent pressure profile can be represented by their combination:

(23) $$\hat{P}\left(x,y,\left\{x^{k}\right\},\left\{y^{k}\right\},t\right)=\sum_{k=1}^{n_{k}}A_{k}\left(x+x^{k}\left(t\right),y+y^{k}\left(t\right)\right)$$

where $\left(x^{k}\left(t\right),y^{k}\left(t\right)\right)$ is the position of the *k*th footprint at time *t*. When only given access to the set of measured pressure approximations of the PSM ${\left\{{\bar{P}}_{ij}\left(t\right)\right\}}_{i=1,\cdots ,n_{r},\;j=1,\cdots ,n_{c}}$ at time *t*, the position of the footprint areas can be computed to match these data as closely as possible. This is achieved by minimising their difference, i.e.,

(24) $${\left\{x^{k},y^{k}\right\}}^{*}\left(t\right):=\min_{\left\{x^{k},y^{k}\right\}}\left\{\sum_{i}\sum_{j}{\left(\hat{P}\left(x_{j},y_{i},\left\{x^{k}\right\},\left\{y^{k}\right\},t\right)-{\bar{P}}_{ij}\left(t\right)\right)}^{2}\right\}$$

where sensor locations $\left(x_{j},y_{i}\right)$ are given by (2). Having solved (24), the overall CoP is then computed using

(25) $$x_{E}\left(t\right)=\frac{{\;\int \int \;}_{A}x\hat{P}\left(x,y,\left\{x^{k}\right\},\left\{y^{k}\right\},t\right)dA}{{\;\int \int \;}_{A}\hat{P}\left(x,y,\left\{x^{k}\right\},\left\{y^{k}\right\},t\right)dA},\;y_{E}\left(t\right)=\frac{{\;\int \int \;}_{A}y\hat{P}\left(x,y,\left\{x^{k}\right\},\left\{y^{k}\right\},t\right)dA}{{\;\int \int \;}_{A}\hat{P}\left(x,y,\left\{x^{k}\right\},\left\{y^{k}\right\},t\right)dA}$$

If ${\left\{A_{k}\left(x,y\right)\right\}}_{k}$ is approximated by high-resolution data (rather than an analytic form), then (25) is replaced by

(26) $$\begin{array}{cc} x_{E}\left(t\right) & =\frac{\sum_{y\in Y}\sum_{x\in X}x\hat{P}\left(x,y,\left\{x^{k}\right\},\left\{y^{k}\right\},t\right)\Delta x\Delta y}{\sum_{y\in Y}\sum_{x\in X}\hat{P}\left(x,y,\left\{x^{k}\right\},\left\{y^{k}\right\},t\right)\Delta x\Delta y},\\ y_{E}\left(t\right) & =\frac{\sum_{y\in Y}\sum_{x\in X}y\hat{P}\left(x,y,\left\{x^{k}\right\},\left\{y^{k}\right\},t\right)\Delta x\Delta y}{\sum_{y\in Y}\sum_{x\in X}\hat{P}\left(x,y,\left\{x^{k}\right\},\left\{y^{k}\right\},t\right)\Delta x\Delta y}. \end{array}$$

The area of the pressure profile may also be known (i.e., the area of a shoe or foot), but the force applied is not. This form of load uncertainty can also be included by generalising (23) to

(27) $$\hat{P}\left(x,y,\left\{x^{k}\right\},\left\{y^{k}\right\},\left\{\alpha^{k}\right\},t\right)=\sum_{k=1}^{n_{k}}\alpha^{k}A_{k}\left(x+x^{k}\left(t\right),y+y^{k}\left(t\right)\right)$$

where $\alpha^{k}$ is the amplitude of the load applied to footprint $A_{k}$, adding $\left\{\alpha^{k}\right\}$ to both sides of (24).

### 3.3. CoP Estimation Using Human Movement Models

On its own, the above-measured footprint optimisation suffers from jittering due to the low-resolution data that it is optimally fitted to. To solve this, an assumption of the movement pattern can be made, which can then be used as a constraint. One assumption is that human motion is typically smooth and follows a predictable pattern. Numerous models for human movement exist and are often represented as constrained minimisation problems. Perhaps the most common models of human movement are minimal jerk [35] or minimal acceleration [36]. It is, therefore, an obvious extension to embed these movement models into the optimisation problem of Section 3.2 to increase their accuracy, especially in the presence of sensor noise. For example, if the minimal acceleration model is employed, the term added to minimisation (24) is

(28) $$\sum_{k=1}^{n_{k}}\int_{0}^{T}\left({\left(\frac{d^{2}x^{k}\left(t\right)}{dt^{2}}\right)}^{2}+{\left(\frac{d^{2}y^{k}\left(t\right)}{dt^{2}}\right)}^{2}\right)dt.$$

## 4. Application Scenarios

To implement the optimisations proposed in Section 3, a set of realistic use-case scenarios is needed to simulate the CoP across multiple time steps so that the error can be minimised by varying the geometry in Section 3.1. For all the optimisations, the CoP errors from the simulation scenarios will be compared to the CoP errors of simulation scenarios with the optimisations implemented. The simulations use the mathematical model proposed in Section 2 by varying the movement and pressure in the profile, P(x,y,t).

The following use-case scenarios are based on common exercises used in balance assessments and commercial balance training platforms, such as the BIODEX BioSway [2]. In static balance tasks, users move their CoP by shifting weight between their feet without moving the position of the feet (their feet form something known as their ‘base of support’, which is the region in which their centre of mass must be kept so that they do not lose balance). The literature uses tasks such as functional reach, where the user must not move their feet (meaning their base of support is fixed) while trying to move their hand and body as close as possible towards a target object [38]. To mimic this weight-shifting task, we have the side weight shift (Section 4.1) and front weight shift scenarios (Section 4.2), which explore the full range of movement from front to back (anterior-posterior) and side to side (medial-lateral), with a fixed base of support. Similar anterior-posterior and medial-lateral movements are used in research to train and/or test balance ability, and these weight shifts are also experienced in exercise games [39,40]. In the case of dynamic balance movements where the user moves their feet to keep their centre of mass within their base of support, we have a balancing task in which the user slides a foot across the platform. This involves a continual change in foot placement and is considered in Section 4.3.

See Appendix A for a description of the parameters used in the following equations. Note that (x,y) can be replaced with subscripts, ij, to make the equation discrete.

### 4.1. Side Weight Shift

**Figure 5:**
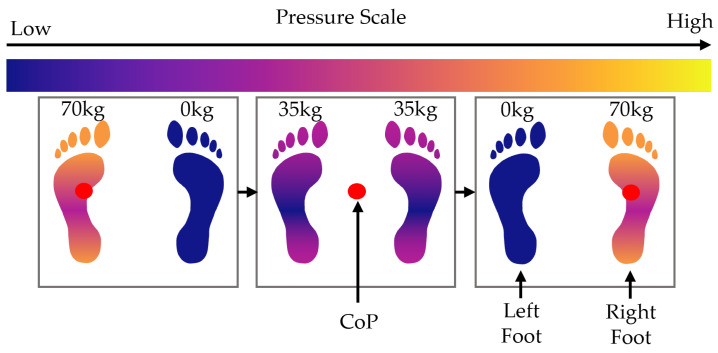
Diagram showing the movement of weight and CoP during the side weight shift scenario.

This movement simulates shifting weight from one foot to the other while keeping the two feet stationary. The CoP moves from the centre of both feet to the left foot, then back to the centre, and then to the right foot and back to the centre again. The movement is shown in Figure 5. The following equation describes the pressure profile that represents this movement over time t∈[0,T]:

(29) $$P\left(x,y,t\right)=\frac{m_{T}gD_{L}\left(x,y\right)}{A\left(x,y\right)T}\left(T-t\right)+\frac{m_{T}gD_{R}\left(x,y\right)}{A\left(x,y\right)T}t.$$

### 4.2. Front Weight Shift

**Figure 6:**
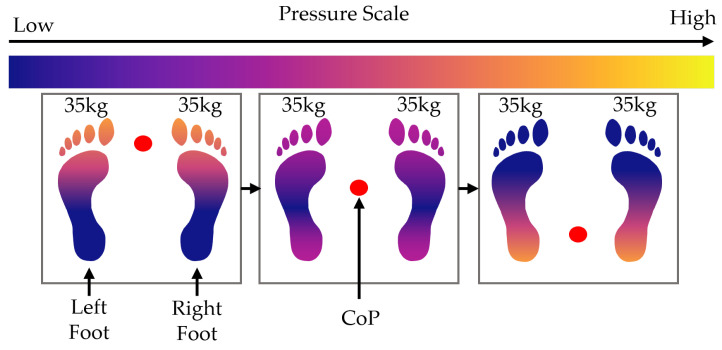
Diagram showing the movement of weight and CoP during the front weight shift scenario.

In this scenario, the user transfers their CoP from the centre of their feet to the toes, back to the centre, to their heels, and back to the centre of the feet again. The size of the foot pressure changes during this simulation, as the surface area on the ground decreases when the user stands on their toes or leans back on their heels. This is illustrated in Figure 6. The following equations describe this pressure movement over time t∈[0,T]:

For the front weight shift, the surface area, A(x,y,t), changes with time, along with the pressure profile $D_{T}\left(x,y,t\right)$, which experiences the same force but a changing surface area; this causes a change in the pressure profile. The area and pressure profile are defined by the following piecewise functions:

(30) $$\bar{A}\left(x,y,t\right)=\left\{\begin{array}{cc} 0 & y<\frac{3h_{m}+h_{f}}{6}-\frac{4h_{f}}{3T}t,\;0\leq t\leq \frac{T}{2}\\ A\left(x,y\right) & y\geq \frac{3h_{m}+h_{f}}{6}-\frac{4h_{f}}{3T}t,\;0\leq t\leq \frac{T}{2}\\ 0 & y\geq \frac{4h_{f}}{3T}t+\frac{3h_{m}-5h_{f}}{6},\;\frac{T}{2}<t\leq T\\ A\left(x,y\right) & y<\frac{4h_{f}}{3T}t+\frac{3h_{m}-5h_{f}}{6},\;\frac{T}{2}<t\leq T \end{array}\right.$$

(31) $${\bar{D}}_{T}\left(x,y,t\right)=\left\{\begin{array}{cc} 0 & y<\frac{3h_{m}+h_{f}}{6}-\frac{4h_{f}}{3T}t,\;0\leq t\leq \frac{T}{2}\\ D_{T}\left(x,y\right) & y\geq \frac{3h_{m}+h_{f}}{6}-\frac{4h_{f}}{3T}t,\;0\leq t\leq \frac{T}{2}\\ 0 & y\geq \frac{4h_{f}}{3T}t+\frac{3h_{m}-5h_{f}}{6},\;\frac{T}{2}<t\leq T\\ D_{T}\left(x,y\right) & y<\frac{4h_{f}}{3T}t+\frac{3h_{m}-5h_{f}}{6},\;\frac{T}{2}<t\leq T \end{array}\right.$$

where the mat height, $h_{m}$, can be computed using

(32) $$h_{m}=\sum_{i=1}^{n_{r}}p_{h_{i}}+c_{h_{i}}.$$

The pressure profile then becomes

(33) $$P\left(x,y,t\right)=\frac{m_{T}g{\bar{D}}_{T}\left(x,y,t\right)}{\bar{A}\left(x,y,t\right)}$$

### 4.3. Foot Slides

**Figure 7:**
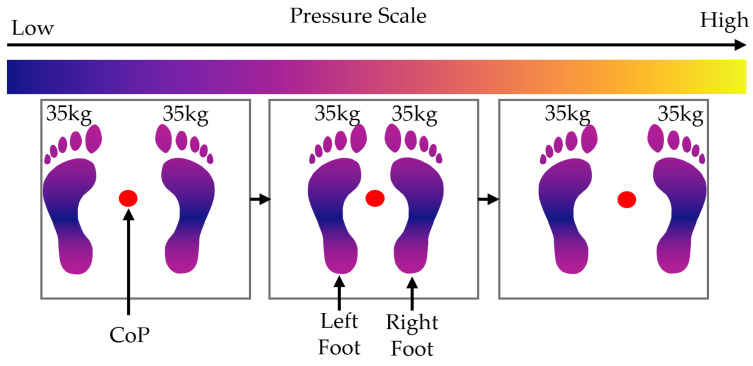
Diagram showing the movement of weight and CoP during the foot slide scenario.

In this scenario, one foot is kept stationary while the other foot slides to the side, increasing the distance between the feet. The moving foot slides outwards and then back inwards to its starting location. Then, the other foot slides outwards and back again. See Figure 7. The following piecewise equation describes the pressure profile that represents this movement over time t∈[0,T]:

(34) $$P\left(x,y,t\right)=\left\{\begin{array}{cc} \frac{m_{T}g}{2A(x,y)}\left(D_{L}\left(x-\left[\frac{2t}{T}\left(l_{x_{e}}-l_{x_{s}}\right)+l_{x_{s}}\right],y\right)+D_{R}\left(x,y\right)\right) & 0\leq t\leq \frac{T}{2}\\ \frac{m_{T}g}{2A(x,y)}\left(D_{L}\left(x,y\right)+D_{R}\left(x-\left[\frac{2t}{T}\left(r_{x_{e}}-r_{x_{s}}\right)+2r_{x_{s}}-r_{x_{e}}\right],y\right)\right) & \frac{T}{2}<t\leq T \end{array}\right.$$

## 5. Methods

This section describes how the model, optimisations, and simulation scenarios described previously are implemented in software to generate results. The optimisation approaches developed in Section 3 minimise the CoP error by either improving the geometry (Section 3.1) or fitting a known footprint (Section 3.2) with respect to the scenarios in Section 4. These are implemented using a set of algorithms, which are found below in Section 5.2 and Section 5.3.

### 5.1. Implementation Details

The simulation software was written in Python 3.13. A set of high-resolution pressure profiles, both of $l_{f}=260$ mm and $w_{f}=103$ mm were imported into the program. The pressure profiles of these high-resolution footprints were scaled to represent a user that weighs $m_{T}=70$ kg. The mat size used for the simulations is 48 cm by 48 cm. The mat geometry of 8 × 8 ($n_{r}=n_{c}=8$) was considered, as it is considered low-cost and quick to manufacture. For a comparison of the results, the 8 × 8 geometry is compared with a high-resolution 512 × 512 ($n_{r}=n_{c}=512$) geometry, which is taken as the “True CoP”. For the sensor parameters, $R_{0}=0.2325\;\Omega$, $k=1.266\times 10^{-8}$, and $R_{d}=465\;\Omega$ were used. To mimic the variability in Velostat force measurements, a constant random noise was added to the force applied to each individual sensor using Numpy’s random number generator with a seed of 38. These random forces ranged from −10 kN to 10 kN and followed a continuous uniform random distribution. Equation (12) was used to compute the pressure of each sensor, and CoP was computed using (2).

The simulation scenarios described in Section 4 were implemented using time steps of size t=0.1 s, from 0≤t≤5, meaning there were 51 time steps in each scenario, all reporting positions of the true CoP, $\left(x_{A}\left(t\right),y_{A}\left(t\right)\right)$, and the estimated CoP, $\left(x_{E}\left(t\right),y_{E}\left(t\right)\right)$. To assess the performance of each optimisation, the percentage error between the actual CoP and estimated CoP for each time step *t* is computed using

(35) $$E_{x}\left(t\right)=100\left(\frac{x_{E}\left(t\right)-x_{A}\left(t\right)}{x_{A}\left(t\right)}\right),\;E_{y}\left(t\right)=100\left(\frac{y_{E}\left(t\right)-y_{A}\left(t\right)}{y_{A}\left(t\right)}\right).$$

The mean percentage error for a scenario is then computed by taking the average of the error across all time steps, mathematically defined as

(36) $$E_{a}=\frac{\sum_{t=0}^{T}\left(\sqrt{E_{x}{\left(t\right)}^{2}+E_{y}{\left(t\right)}^{2}}\right)}{T}.$$

The mean percentage errors $E_{a}$ for each individual scenario can then be averaged together to find the average error score for an optimisation method.

### 5.2. Optimal PSM Geometry Algorithm

To efficiently implement the geometry optimisation approach in Section 3.1 requires minimising the search space used within optimisation (20). This is achieved by defining a minimal set of possible values for each parameter in θ. For example, let ${\underline{c}}_{w_{1}}$ and ${\bar{c}}_{w_{1}}$ be the minimum and maximum values of $c_{w_{1}}$ so that $c_{w_{1}}\in \left[{\underline{c}}_{w_{1}},{\bar{c}}_{w_{1}}\right]:=C_{w_{1}}$. Defining similar sets for all parameters leads to the overall parameter space

(37) $$\Theta :=C_{w_{1}}\times \cdots \times C_{w_{n_{c}}}\times C_{h_{1}}\times \cdots \times C_{h_{n_{r}}}\times P_{w_{1}}\times \cdots \times P_{w_{n_{c}}}\times P_{h_{1}}\times \cdots \times P_{h_{n_{r}}}.$$

The solution can then be computed using a simple brute force search, as set out in Algorithm 1.
**Algorithm 1** Optimal PSM geometry
     **Input:** Parameter search space Θ     **Output:** Optimal parameter vector $\theta^{*}$
1:**for** i=1,⋯,|Θ| **do**2:    $\theta \leftarrow \Theta_{i}$       ▹$\Theta_{i}$ is *i*th element of Θ3:    $J_{i}\leftarrow J\left(\theta \right)$, where J(θ) is given by (20) and $\tilde{P}\left(x,y,t\right)$ is given by (29)4:    $J_{i}\leftarrow J_{i}+J\left(\theta \right)$, where J(θ) is given by (20) and $\tilde{P}\left(x,y,t\right)$ is given by (33)5:    $J_{i}\leftarrow J_{i}+J\left(\theta \right)$, where J(θ) is given by (20) and $\tilde{P}\left(x,y,t\right)$ is given by (34)6:**end for**7:$i^{*}\leftarrow \min_{i}\left\{J_{i}\right\}$8:$\theta^{*}\leftarrow \theta_{i^{*}}$,9:**return** $\theta^{*}$


Algorithm 1 is a brute-force search that iterates over every possible combination in the parameter space set out in Θ. This loop begins on line 1 of the iteration. Within the loop, θ is set to the *i*th element of Θ. J(θ) is then computed using the current θ in Equation (20) and the side weight shift simulation scenario equation described by (29), which is then given to $J_{i}$. In line 4 of Algorithm 1, J(θ) is computed again, but for the front weight shift scenario (33), with the result being added to the current state of $J_{i}$. Similarly, line 5 computes J(θ) but for the front weight shift scenario in Equation (34), with this result also being added to $J_{i}$. These summations mean that the final $J_{i}$ is the sum of J(θ) for each individual simulation scenario. After the loop finishes computing every $J_{i}$, line 7 then selects the minimum $J_{i}$ as $i^{*}$. Lines 8 and 9 return $\theta^{*}$, which is the optimal mat geometry that produced the minimum cost $J_{i^{*}}$.

### 5.3. CoP Estimation Using Measured Footprint Algorithm

Algorithm 2 summarises the CoP estimation approach described in Section 3.2. This algorithm computes the best match between a known footprint and the data generated experimentally by the PSM. The set θ, containing the known PSM geometry, is required, as well as a set of $n_{k}$ previously measured footprints ${\left\{A_{k}\left(x,y\right)\right\}}_{k=1,\ldots ,n_{k}}$. The optimisation (38) can be solved using a brute force search, i.e., the expression in parenthesis is evaluated for a suitably high-resolution set of footprint positions, $\left\{\left(x^{k},y^{k}\right)\right\}$, and the minimum value taken. This set of possible footprint positions at time *t* can encompass the entire mat or can be limited to an area surrounding their positions at the previous sample instant to reduce the search space.
**Algorithm 2** Measured Footprint Optimisation
     **Input:** Experimental pressure values $\left\{{\bar{P}}_{ij}\left(t\right)\right\}$ provided by the PSM at time *t*     **Input:** Set of measured footprints ${\left\{A_{k}\left(x,y\right)\right\}}_{k=1,\ldots ,n_{k}}$     **Input:** PSM geometry parameters θ     **Output:** CoP estimate $\left(x_{E}\left(t\right),y_{E}\left(t\right))\right)$ at time *t*
1:(38)$$\left(x^{k}{\left(t\right)}^{*},y^{k}{\left(t\right)}^{*}\right)\leftarrow \min_{\left\{x^{k},y^{k}\right\}}\left\{\sum_{i}\sum_{j}{\left(\sum_{k=1}^{n_{k}}A_{k}\left(x_{j}+x^{k}\left(t\right),y_{i}+y^{k}\left(t\right)\right)-{\bar{P}}_{ij}\left(t\right)\right)}^{2}\right\}$$        ▹ Compute footprint locations (by combining (24) with (23))2:(39)$$\begin{array}{cc} \; & x_{E}\left(t\right)\leftarrow \frac{{\;\int \int \;}_{A}x\sum_{k=1}^{n_{k}}A_{k}\left(x+x^{k}{\left(t\right)}^{*},y+y^{k}{\left(t\right)}^{*}\right)dA}{{\;\int \int \;}_{A}\sum_{k=1}^{n_{k}}A_{k}\left(x+x^{k}{\left(t\right)}^{*},y+y^{k}{\left(t\right)}^{*}\right)dA},\; \end{array}$$(40)$$\begin{array}{cc} \; & y_{E}\left(t\right)\leftarrow \frac{\sum_{y\in Y}\sum_{x\in X}y\sum_{k=1}^{n_{k}}A_{k}\left(x+x^{k}{\left(t\right)}^{*},y+y^{k}{\left(t\right)}^{*}\right)\Delta x\Delta y}{\sum_{y\in Y}\sum_{x\in X}\sum_{k=1}^{n_{k}}A_{k}\left(x+x^{k}{\left(t\right)}^{*},y+y^{k}{\left(t\right)}^{*}\right)\Delta x\Delta y} \end{array}$$              ▹ Compute CoP (by combining (26) with (23)3:**return** $\left(x_{E}\left(t\right),y_{E}\left(t\right))\right)$


## 6. Results

In this section, we present diagrams of the default track geometry and of the optimised geometry, which was generated using the algorithm detailed in Section 5.2. Along with the diagrams, we also present our results, which were produced using the approach outlined in Section 5.

**Figure 8:**
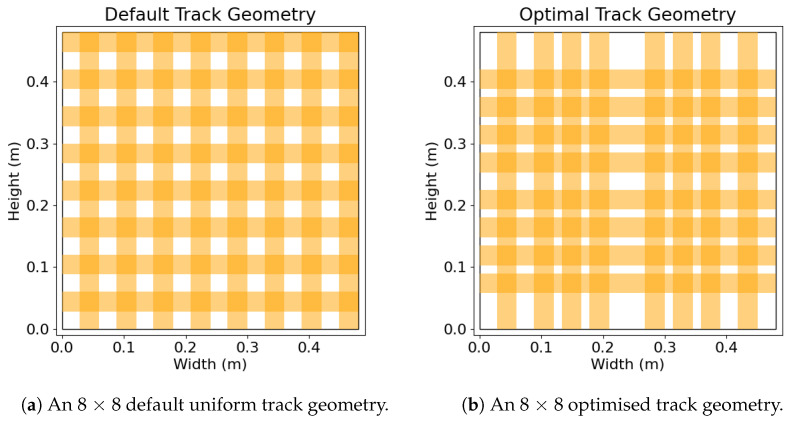
Figures showing the track geometries used in the simulations, where (**a**) is the default standard uniform track layout, while (**b**) is a track layout generated using our algorithm in Section 5.2.

The optimal geometry shown in Figure 8 reveals a higher density of tracks where the foot profiles spend most of their time in the simulations, which is to be expected. Less resolution is needed in areas of the mat that are seldom used, whereas a higher density of sensors can be applied to high-use areas.

**Figure 9:**
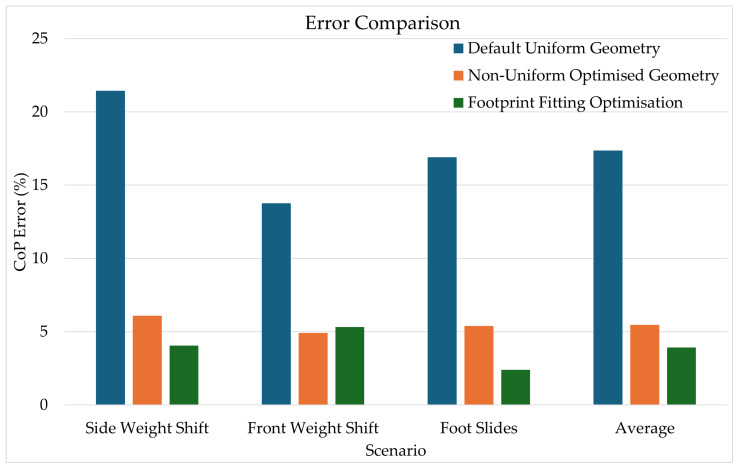
A graphical representation of the results in Table 1.

From Table 1 and Figure 9, the geometry optimisation errors are from running the scenarios using the optimal track layout shown in Figure 8b. Individually, the geometry optimisation and known foot profile work very well and decrease the average error from 17.37% to 5.47% and 17.37% to 3.93%, respectively, reducing the error by more than 10%. Both the geometry optimisation and known footprint yielded a particularly large improvement when improving the side weight shift scenario, decreasing a 21.44% base error to 6.09% and 4.05%, respectively. Note that the results for the measured footprint optimisation (Section 3.2) do not use the human smooth movement assumption described in Section 3.3.

## 7. Discussion

Within the optimal geometry computation (Section 3.1), millions of layout configurations are possible, even for a low-resolution 8 × 8 grid layout. To reduce computational load, it was observed that data profiles that are symmetric in the x- and/or y-axis will give rise to optimal layouts that are also symmetric in these axes. This enables the optimisation to be restricted to consider purely symmetrical layouts, which reduces the search space from millions of geometries to a few hundred.

In this case, the program takes only a couple of minutes to find the optimal geometry rather than hours to days. In the case of higher resolutions, the number of possible layouts exponentially increases, which also exponentially increases the time required to find the optimal mat geometry. Note that the optimal geometries were found using a brute force combinatorial search, which is the least efficient method for minimising the problem. Other techniques for minimisation problems could be explored to speed up how long it takes to find a solution, such as the Nelder-Mead method [41]. An advantage of the optimal geometry method is that once the best geometry has been found, the design can be implemented into a physical PSM, and no further computations are needed. Another advantage is that it generates a globally optimal solution.

The measured footprint (Section 3.2) method can be implemented using the standard CoP measurement as a starting position. This means it can be implemented efficiently in real time. When observing an animated heat map that displayed how the fitted profiles moved during the simulation scenarios, it was noticed that there was random jittering, which likely negatively impacted the CoP error. The human model movement optimisation in Section 3.3 could significantly reduce the jittering, as it smooths out the movement, potentially further reducing the CoP error.

A key consideration for constructing a PSM is the cost, which our mathematical model accounts for, as it was based on a low-cost PSM design (as seen in [27]). The piezoresistive material Velostat is a readily available, low-cost, off-the-shelf component. Multiplexers of up to 16 channels are also low-cost and readily available, along with MCUs and passive components such as resistors. Our optimisations can also be adapted to directly minimise manufacturing cost, which can be achieved by extending the costs J(θ) in Section 3.1 to contain weights, $W_{ij}$, which embed the financial cost of the *i*th row and *j*th column. The goal would then be to attempt to achieve the same accuracy as a uniform layout but with fewer sensors using an optimal layout. This optimisation would allow designers to save on manufacturing time and component cost while achieving the same CoP error that they would have had with a higher resolution uniform layout.

In practice, when running physical tests using the optimal geometry, there are other noise sources that can influence the results that are not yet accounted for in the simulation, such as crosstalk noise between sensors [27,31]. Crosstalk is an issue that can be solved by hardware [42] or through an algorithm [31,34]. Crosstalk removal algorithms are computationally expensive, making these algorithms challenging to implement for real-time systems with a resolution greater than or equal to $n_{c}=n_{r}=16$ [27]. However, as our optimisations enhance the CoP accuracy of low-resolution designs ($n_{c}=n_{r}=8$), real-time crosstalk removal becomes practical. Other known variability issues within practical PSMs are the material properties of piezoresistive materials: creep and hysteresis. Creep and hysteresis can be accounted for with mathematical models that compensate for these effects [32]. Non-repeatability is another variable, and so is each sensor behaving differently under the same load despite the materials and design being the same [29,32,43]. Since crosstalk, hysteresis, and creep have solutions that can minimise their effects, they are not included in the model. The behaviour of the sensors taking differing measurements is considered in the simulations through uniformly distributed random noise, as detailed in Section 5.1.

## 8. Conclusions and Future Work

This paper developed the first generalised model of a low-cost PSM (to the authors’ knowledge) that can be used to create and test optimisations to improve CoP accuracy. To demonstrate the model, three optimisations were proposed, and two were implemented and simulated, with each optimisation more than halving the CoP measurement error when used on its own. The enhanced accuracy makes low-cost PSMs a more viable option for home-based balance measurement, which could be made even better when applying de-noising algorithms.

Future work will first include applying the approaches developed in this paper onto a physical PSM to test the simulation results in practice. Multiple subjects will perform the movements described in Section 4, and statistic analysis will be conducted on the resulting CoP measurements to determine the mean, variance, and statistical significance of the CoP accuracy improvement for each scenario.

The work in this paper also opens the door to testing more algorithms, such as the human movement model assumption in Section 3.3. Other future work includes improving the simulation model by adding noise, such as crosstalk [31]. The Python scripts used to simulate and run these models could also be integrated into a GUI that allows designers to produce their own geometry optimisations based on their use case requirements. This would give designers an easy way to generate more accurate PSMs without adding additional cost to their system.

As our optimisations are designed to improve low-cost PSMs, such PSMs could be used in home-based balance monitoring and training, making data-driven feedback-based training accessible to anyone. Coaches and physical therapists could also use these PSMs to aid in improving the performance of their clients. Outside the realm of enhancing human performance, optimised, low-cost PSMs could be used for the following: prevent discomfort in hospital patients [20], an input to interactive systems such as video games [44,45], a way to control robots or other industrial processes [46], or help control bipedal robots through CoP measurement [47]. In conjunction with AI, such mats could potentially be used to identify users with a significant risk of falling [22].

## Figures and Tables

**Table 1:** Percentage errors for an 8 × 8 standard grid geometry with and without optimisations. The percentage errors between the “True CoP” (from a 512 × 512 grid) and the 8 × 8 grid are given.

Scenario	Default Uniform Geometry	Non-Uniform Optimised Geometry	Footprint Fitting Optimisation
Side Weight Shift	21.44%	6.09%	4.05%
Front Weight Shift	13.77%	4.92%	5.33%
Foot Slides	16.91%	5.40%	2.40%
Average	17.37%	5.47%	3.93%

## Data Availability

The original data and the code used to generate this data has been made available in ePrints Soton at DOI: 10.5258/SOTON/D3384.

## Abbreviations

The following abbreviations are used in this manuscript:

PSM	Pressure-Sensitive Mat
CoP	Centre of Pressure
MCU	Microcontroller
ADC	Analogue-digital converter
GPIO	General-purpose input/output
IMU	Inertial measurement unit
AI	Artificial Intelligence

## Appendix A. List of Variables

- *t*: Time (s).
- *m*: Mass of a load (kg).
- (x,y): Co-ordinates on the surface (m).
- $\left(x_{A},y_{A}\right)$: Actual co-ordinates of the CoP (m).
- $\left(x_{E},y_{E}\right)$: Estimated co-ordinates of the CoP (m).
- $\left(E_{x},E_{y}\right)$: Percentage error between the actual and estimated CoP (%).
- $\left(x_{j},y_{i}\right)$: Sensor midpoint co-ordinates at index *j* and *i* (m).
- $n_{r}$: Number of rows (unitless).
- $n_{c}$: Number of columns (unitless).
- $p_{h_{k}}$: Pitch heights for the rows, at index *k* (m).
- $p_{w_{k}}$: Pitch widths for the columns, at index *k* (m).
- $c_{h_{k}}$: Conductor track heights for the rows, at index *k* (m).
- $c_{w_{k}}$: Conductor track widths for the columns, at index *k* (m).
- P(x,y): A continuous pressure profile (Pa).
- $\hat{P}\left(x,y,t\right)$: A set of pressure profiles or a pressure profile that varies with time (Pa).
- $P_{ijs}$: Applied pressure of a segment *s* on a sensor positioned at ij (Pa).
- $P_{ij}$: Total applied pressure of a sensor positioned at ij (Pa).
- ${\left\{{\bar{P}}_{ij}\right\}}_{i=1,\ldots ,n_{r},j=1,\ldots ,n_{c}}$: A set of approximations of $P_{ij}$ (Pa).
- $A_{ijs}$: Surface area of a segment *s* of a sensor positioned at ij ($m^{2}$).
- $A_{ij}$: Total surface area of a pressure sensor positioned at ij ($m^{2}$).
- $R_{ijs}$: Resistance of a segment *s* on a sensor positioned at ij (Ω).
- $R_{ij}$: Total resistance of a sensor positioned at ij (Ω).
- $R_{d}$: The resistance of the potential divider resistor (Ω).
- $R_{0}$: Initial resistance of a 1m by 1m unloaded sensor (Ω).
- κ: Slope of the pressure-resistance curve (unitless).
- *b*: number of bits of the MCU’s ADC (bit).
- $V_{cc}$: Input voltage to the sensors (V).
- $V_{ij}$: Voltage of a sensor at position ij (V).
- ${\bar{V}}_{ij}$: Quantised $V_{ij}$ (V).
- *T*: Total time of a pressure profile or an appended set of profiles (s).
- θ: A vector of pitch widths and heights, and conductor track widths and heights (m).
- J(θ): The error 2-norm used to optimise the PSM for minimal CoP error (unitless).
- $n_{k}$: Number of footprints within a set of known footprints (unitless).
- ${\left\{A_{k}\left(x,y\right)\right\}}_{k=1,\ldots ,n_{k}}$: A set of known, previously measured footprints (Pa).
- $\left\{\left(x^{k},y^{k}\right)\right\}$: Position of known footprint *k* (m).
- $\alpha^{k}$: Amplitude of the load applied to a footprint *k* (unitless).
- $m_{T}$: Total mass of the user (kg).
- *g*: Gravitational acceleration constant ($\frac{m}{s^{2}}$).
- $w_{f}$: Width of foot (m).
- $h_{f}$: Height of foot (m).
- $w_{m}$: Width of mat (m).
- $h_{m}$: Height of mat (m).
- $D_{L_{ij}}$: Total pressure at sensor ij on the default left foot pressure profile, where $m=\frac{m_{T}}{2}$ (Pa).
- $D_{R_{ij}}$: Total pressure at sensor ij on the default right foot pressure profile, where $m=\frac{m_{T}}{2}$ (Pa).
- $D_{T_{ij}}$: Total pressure at sensor ij on the default pressure profile $D_{T}=D_{L}+D_{R}$. (Pa).
- $l_{x_{s}}$: Left foot starting position *x* co-ordinate (m).
- $l_{x_{e}}$: Left foot ending position *x* co-ordinate (m).
- $r_{x_{s}}$: Right foot starting position *x* co-ordinate (m).
- $r_{x_{e}}$: Right foot ending position *x* co-ordinate (m).
